# Supplementary material for: Janus nanoparticles targeting extracellular polymeric substance achieve flexible elimination of drug-resistant biofilms
Source: Nat Commun. 2023 Aug 23;14:5132. doi: 10.1038/s41467-023-40830-9 (PMC10447547; doi:10.1038/s41467-023-40830-9)
Supplement: Supplementary file 1 — Supplementary Information [file 41467_2023_40830_MOESM1_ESM.pdf]

Supplementary Information

Janus nanoparticles targeting extracellular polymeric substance achieve flexible elimination of drug-resistant biofilms

Zhiwen Liu,[1,2,3,†] Kangli Guo,[1,2,3,†] Liemei Yan,[1,2,3] Kai Zhang,[1,2,3] Ying Wang,[1,2,3] Xiaokang Ding,[1,2,3] Nana Zhao,[1,2,3]* Fu-Jian Xu[1,2,3]*

[1]State Key Laboratory of Chemical Resource Engineering, Beijing University of Chemical Technology, Beijing, 100029, China
[2]Key Laboratory of Biomedical Materials of Natural Macromolecules (Beijing University of Chemical Technology), Ministry of Education, Beijing 100029, China
[3]Beijing Laboratory of Biomedical Materials, Beijing University of Chemical Technology, Beijing, 100029, China

* To whom correspondence should be addressed
Email: xufj@mail.buct.edu.cn, zhaonn@mail.buct.edu.cn
†These authors contributed equally to this work.

Table of Contents

Supplementary Methods 3

Supplementary Figures and Discussion 7

Supplementary References...20

Supplementary Methods

Synthesis of bismuth selenide (BSe) nanosheets. BSe nanosheets were prepared by a solution method following the previous work with some modifications[1,2]. In brief, the Se source solution was prepared by dissolving 5 mg of Se powder and 0.1 g of $NaBH_4$ in 2 mL of water in an ice-water bath. Subsequently, 0.08 g of PVP dissolved in 4 mL of ethylene glycol was mixed with the Se source solution, which was followed by the addition of 50 μL of $BiCl_3$ solution (0.1 M in 1.8 M HCl). Then, the mixture was hydrothermally treated for 12 h at 160 °C. The final production was purified by centrifugation (23478 × *g*, 20 min) and washed with deionized water for 3 times.

For Cy5.5 labeling, 20 mg of SH-PEG-NH_2 was mixed with 5 mL of BSe aqueous solution (1 mg/mL) and kept stirring for 12 h. The nanoparticles were collected by centrifugation and then dispersed in water. 10 μL of Cy5.5-NHS solution (5 mg/mL) was added to 1 mL of the BSe nanoparticles solution (5 mg/mL). Thereafter, Cy5.5-labeled BSe were collected by centrifugation (23478 × *g*, 20 min) and washed with ethanol until the supernatant was colorless.

Synthesis of aminated dextran (Dex-NH_2). Dex-NH_2 was prepared according to our previous report[3]. Briefly, 0.5 g of dextran was dissolved in 3.5 mL anhydrous DMSO and degassed by N_2 for 10 min. Then 0.5 g of CDI in 1.5 mL of anhydrous DMSO was added dropwise. The obtained solution was degassed by bubbling nitrogen for 10 min and kept at room temperature for 4 h. Then, 1.5 mL of ethylenediamine (ED) was injected into the above solution under vigorous stirring. Afterwards, the mixture was stated at room temperature under nitrogen environment for 48 h. The final product (Dex-NH_2) was obtained after dialysis (MWCO 3500) at room temperature for 48 h and lyophilization.

Characterization. The obtained CS-BSe and Dex-BSe were characterized by Transmission electron microscopy (TEM, HT-7800, Hitachi, Japan), scanning electron microscope (SEM, Zeiss Supra 55), thermal gravimetric analysis (TGA, Tarsus TG 209 F3, Netzsch, Germany), confocal laser scanning microscopy (CLSM, TCS SP8, Leica, Mannheim, Germany), and particle size and zeta potential measurements (Zetasizer Nano ZS, Malvern Instruments, Southborough, MA). For TEM specimen preparation, the samples were dropped onto a Formvar-covered copper grid, followed by drying naturally. TGA was performed at a heating rate of 10 ^{o}C/min under an air atmosphere. Dextran-NH_2 was characterized by nuclear magnetic resonance (NMR) spectroscopy (Bruker ARX, 400 MHz). The temperature measurement and thermal images were obtained using an infrared thermal imaging instrument (FLIR Systems Inc., Ohio, USA) at each time point. The near-infrared (NIR) laser was performed using an 808 nm continuous wave diode laser (Daheng New Epoch Technology, Inc., Beijing, China).

Photothermal effect of BSe, CS-BSe and Dex-BSe in vitro. To test the photothermal property, 100 μL of BSe, CS-BSe or Dex-BSe aqueous solutions with the BSe concentration of 410, 205, 102, 51, and 26 μg/mL, respectively, were added into a 96-well plate, and then irradiated by an 808 nm NIR laser at a power density of 1.0 W/cm^2 for 5 min. An IR thermal camera was used to record the temperature every 30 seconds.

To study the photothermal stability, Dex-BSe aqueous solution (400 μg/mL) was irradiated for 5 min, followed by naturally cooling to room temperature, and this cycle was repeated five times. In order to test the photothermal property of nanoparticles in different medium, 100 μL of solutions of BSe, CS-BSe and Dex-BSe in different medium (PBS and PBS with 10^5 CFU *S. aureus*) with the same BSe concentration of

205 μg/mL were added in 96-well plates respectively, and then irradiated by a NIR laser at 808 nm with a power density of 1.0 W/cm^2 for 5 min. An IR thermal camera was used to record the temperature every 30 seconds.

In vitro cytotoxicity. The cytotoxicity of BSe, CS-BSe, and Dex-BSe were assessed in L929 cell line by MTT assay. L929 cells were seeded on 96-well plates at a density of 1 × 10^4 cells per well and incubated overnight at 37 ºC. Then BSe, CS-BSe, and Dex-BSe solutions with different BSe concentrations (410, 205, 102, 51, and 26 μg/mL) were added. After co-cultured for 24 h, 10 μL of MTT (5 mg/mL in PBS) was added to each well and incubated for 4 h, followed by PBS rinse. Finally, 100 μL of DMSO was added to dissolve the produced formazan crystals. The absorbance of final product formazan at 570 nm was measured by Bio-Rad Model 680 Microplate Reader (UK).

Hemolysis assay. 1 mL of whole blood collected from Sprague–Dawley rats was suspended in 10 mL of PBS. The mixture was centrifuged at 956 × *g* for 15 min to separate red blood cells (RBCs). The RBCs were then washed with PBS until the supernatant was clear. Subsequently, the RBCs were resuspended in PBS at the volume concentration of 4%. 100 μL of Dex-BSe solution with different concentrations (64-2048 μg/mL) was added in 100 μL of RBC suspension in PBS (4%). The final volume concentration of RBCs was 2%. RBCs treated with deionized water and PBS were used as the positive and negative controls, respectively. The resultant RBC suspension were cultured at 37 °C for 3 h and then centrifuged at 956 × *g* for 15 min. The absorbance of the supernatant was measured at 545 nm. The hemolysis ratio was calculated using the following equation:

$$\text{Hemolysis ratio (\%)} = \frac{OD_{test} - OD_{neg}}{OD_{pos} - OD_{neg}} \times 100\ \%$$

where OD_{test}, OD_{neg}, and OD_{pos} are the OD values of samples, negative control, and positive control, respectively.

MRSA infection mouse model. To establish the infection mouse model, female Balb/c mice (6-8 weeks, 16-20 g) were anesthetized with isoflurane. A suspension of MRSA (10^8 CFU/mL, 100 μL) was injected into the left rear thigh muscle. After 24 h, the MRSA-infected abscess was formed.

In vivo imaging and biodistribution of nanoparticles. Cy5.5-labeled Dex-BSe and CS-BSe nanoparticles (5.0 mg/mL) were intravenously injected into the MRSA-infected mice, respectively. At the predetermined time points (0, 2, 4, 6, 8, 10, 12, and 24 h), fluorescent images were obtained employing a Xenogen IVIS Spectrum (Caliper Life Sciences, America). Subsequently, the mice were sacrificed and the major organs (heart, liver, spleen, lung, and kidney) and the infected tissues were extracted for fluorescence imaging.

Supplementary Figures and Discussion

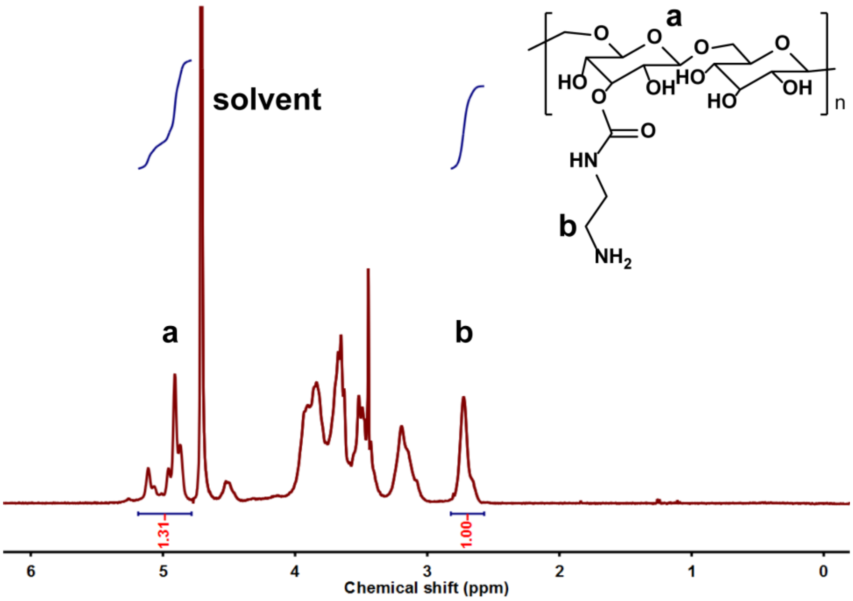

Supplementary Fig. 1 NMR characteriaztion of Dex-NH_2. Typical ^{1}H NMR spectrum of Dex-NH_2 (in D_2O).

The representative structure of Dex-NH_2 was characterized by ^{1}H NMR spectrum as shown in Supplementary Fig. 1. The chemical shift from 4.87 to 5.10 ppm was associated with the methylidyne protons (a, O-CH-O) of glucose units of dextran. The signals at $\delta = 2.90$ ppm correspond to the methylene protons adjacent amine groups (b, CH_2-NH_2). Based on the area ratio of peak a (corresponding to the amount of total glucose units of Dex-NH_2) and peak b (corresponding to the twice the amount of aminated glucose units of Dex-NH_2), it was calculated that about 2.6 (1.31/(1.0/2)) glucose units of Dex-NH_2 possess one amino group.

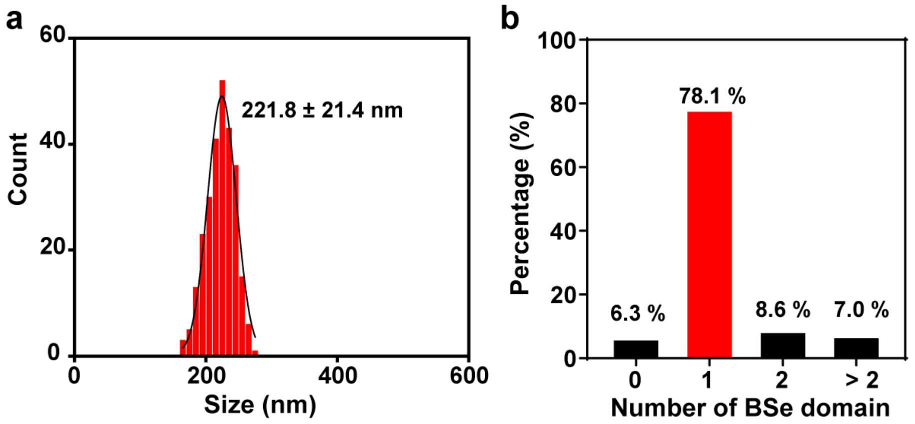

Supplementary Fig. 2 Size distribution and morphology analysis of Janus Dex-BSe nanoparticles. a Size distribution of Dex-BSe nanoparticles. **b** Statistical results of dextran domain loaded with different numbers of BSe domains. Source data are provided as a Source Data file.

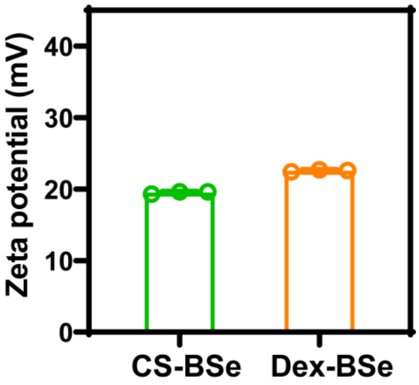

Supplementary Fig. 3 Zeta potential of Dex-BSe and CS-BSe nanoparticles. Data are presented as mean values ± SD (n = 3 independent samples). Source data are provided as a Source Data file.

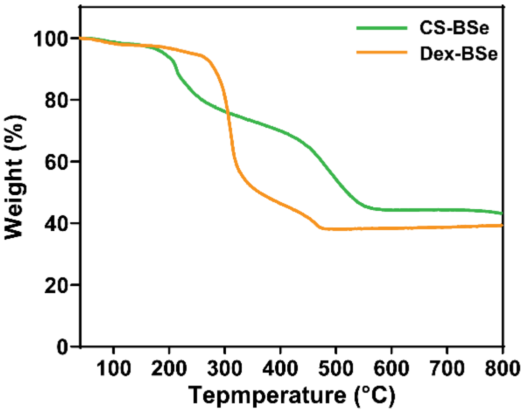

Supplementary Fig. 4 Thermogravimetric analysis of CS-BSe and Dex-BSe nanoparticles. Source data are provided as a Source Data file.

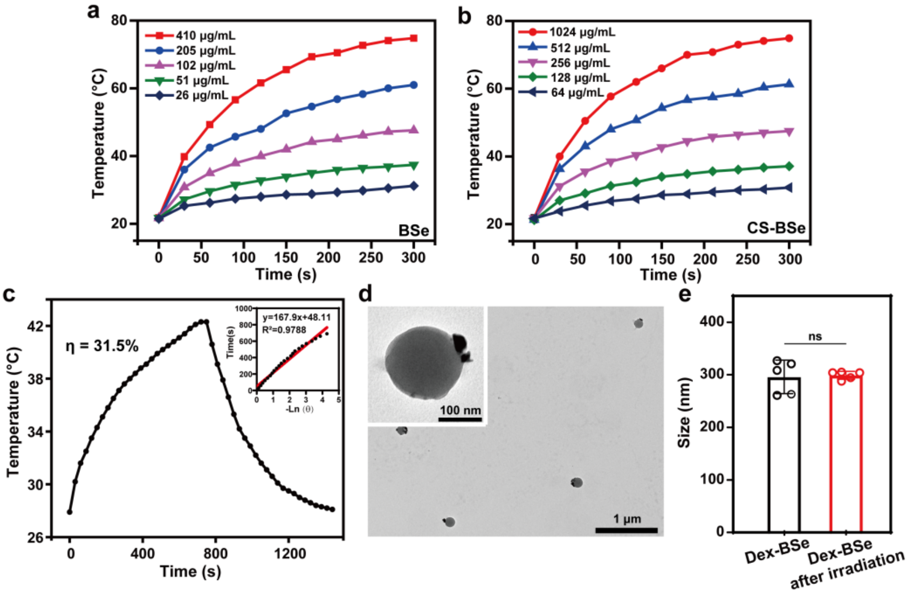

Supplementary Fig. 5 Photothermal properties of nanoparticles. Photothermal curves of BSe (**a**) and CS-BSe (**b**) aqueous solutions with different concentrations under 808 nm laser irradiation (1 W/cm^2, 5 min). **c** Photothermal effect and cooling process of Dex-BSe aqueous solution under 808 nm laser irradiation (1.0 W/cm^2). **d** TEM image of Dex-BSe after 808 nm laser irradiation (1 W/cm^2, 5 min). Experiments were repeated three times independently with similar results. **e** Hydrodynamic particle size of Dex-BSe during five heating and cooling cycles. Data are presented as mean values ± SD (n = 5 independent samples). Statistical significance was calculated by two-tailed Student's t-test. Source data are provided as a Source Data file.

Calculation of the photothermal conversion efficiency. The photothermal conversion efficiency (η) of the Dex-BSe was calculated according to the reported method[4]. Under continuous laser irradiation, the temperature of the Dex-BSe aqueous solution was recorded, until the solution had reached a steady-state temperature at 720 s. The photothermal conversion efficiency (η) of Dex-BSe was calculated via the following formula:

$$\eta = \frac{hS(T_{\max} - T_{\text{surr}}) - Q_{\text{dis}}}{I(1 - 10^{-A_{808}})} \quad (1)$$

Where, h represents the heat transfer coefficient. S is the surface area of the quartz sample cell. $T_{\max}$ and T_{surr} are the maximum steady-state temperature (42.3 °C) and the surrounding temperature of the environment (28.1 °C), respectively. Q_{dis} is the heat dissipation from the light absorbed by the solvent and the quartz sample cell,

which is measured to be 0.082 W. I is the incident laser power (1.0 W·cm^{-2}), and A_{808} is the absorbance intensity of the sample at 808 nm (0.867).

The value of hS was derived from Equation (2):

$$\tau_s = \frac{\sum_i m_i C_{p,i}}{hS} \quad (2)$$

$$\theta = \frac{T - T_{\text{surr}}}{T_{\text{max}} - T_{\text{surr}}} \quad (3)$$

$$t = -\tau_s \ln\theta \quad (4)$$

where τ_s is the time constant for heat transfer of the system which is determined to be τ_s = 167.9 s. The m is 1.0 g and the C_p is 4.2 J/g. So the $hS(T_{\text{max}}-T_{\text{surr}})$ is determined as 0.36 W. According to the obtained data and Equation (1), the photothermal conversion efficiency of Dex-BSe was determined to be ~31.5%.

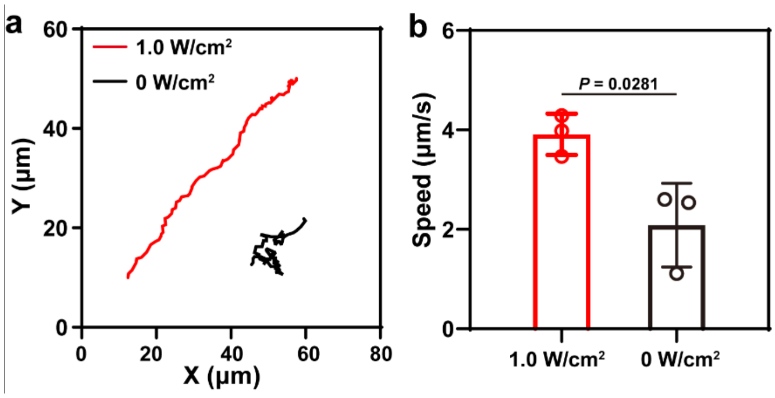

Supplementary Fig. 6 Movement behavior of Dex-BSe nanoparticles. a Trajectories of Dex-BSe in PBS in the absence and presence of NIR laser irradiation (1.0 W/cm^2, 30 s). **b** The average velocity of Dex-BSe at different conditions. Data are presented as mean values ± SD (n = 3 independent samples). Statistical significance was calculated by two-tailed Student's t-test. Source data are provided as a Source Data file.

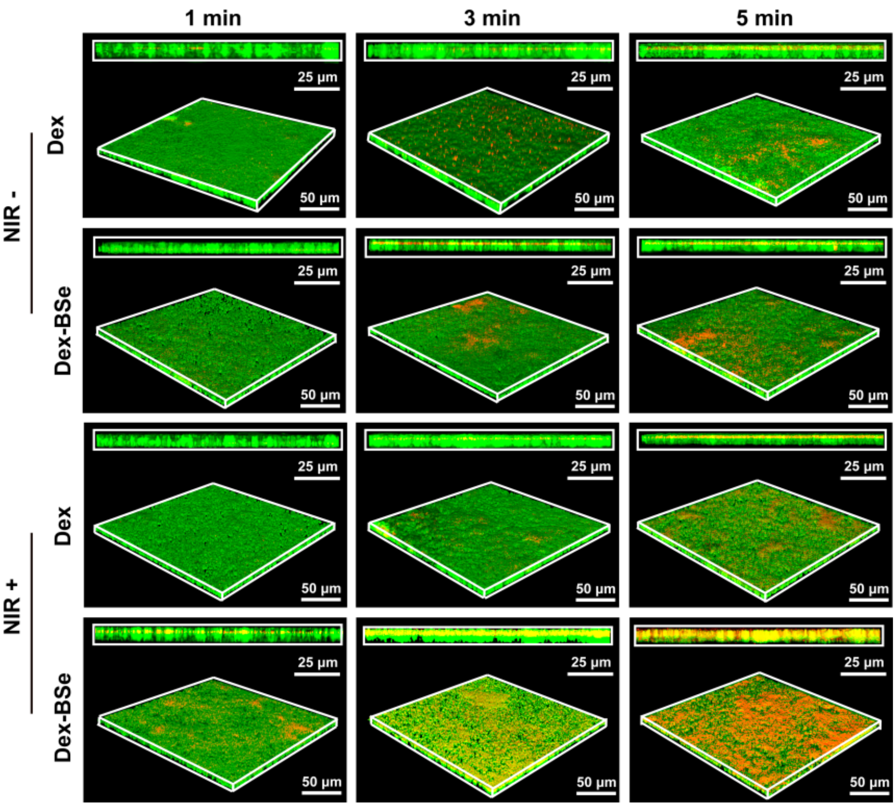

Supplementary Fig. 7 Biofilm penetration of nanoparticles with and without NIR light irradiation. 3D CLSM images and corresponding z-stack images of *S. aureus* biofilms treated with Cy5.5-labeled Dex and Dex-BSe at Dex concentration of 307 μg/mL in the presence or absence of NIR light (808 nm, 1.0 W/cm^2) irradiation for 1, 3, and 5 min. Green: live bacteria, Red: Cy5.5-labeled nanoparticles.

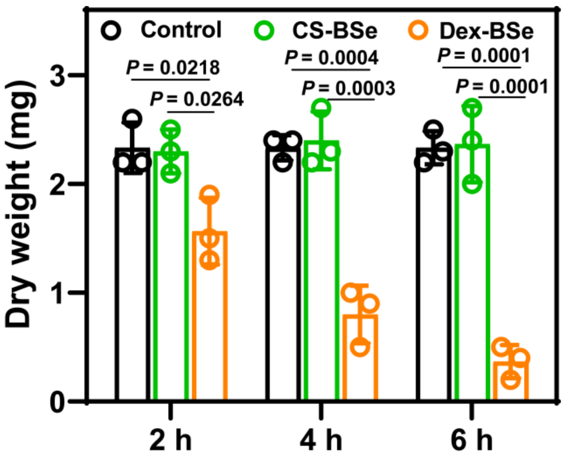

Supplementary Fig. 8 Mass of biofilms after different treatments. Data are presented as mean values ± SD (n = 3 independent samples). Statistical significance was calculated by one-way ANOVA using the Tukey post-test. Source data are provided as a Source Data file.

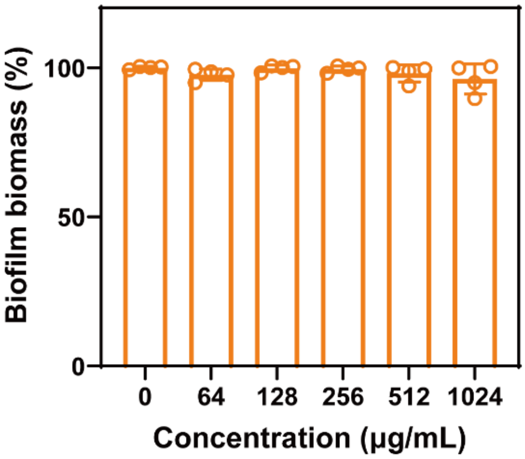

Supplementary Fig. 9 Quantitative analysis of the crystal violet-stained *E. coli* biofilms treated with Dex-BSe at different concentrations. Data are presented as mean values ± SD (*n* = 4 independent samples). Source data are provided as a Source Data file.

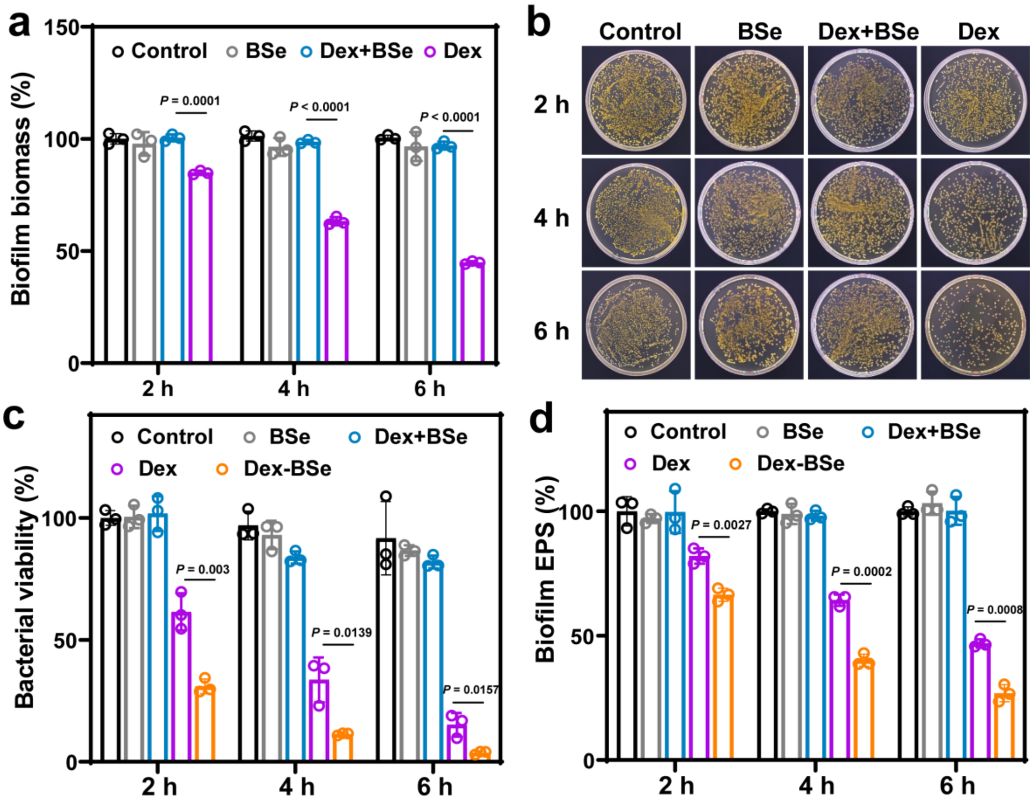

Supplementary Fig. 10 Biofilm dispersion effect of different nanoparticles. a Quantitative analysis of the crystal violet-stained biofilms treated with BSe, Dex+BSe, and Dex at different incubation times. **b** Representative photographs of corresponding *S. aureus* colonies after different treatments. **c** Viability of *S. aureus* biofilms after different treatments determined by a typical plate counting method. **d** Quantitative analysis of the matrix of EPS after treatments with BSe, Dex+BSe, Dex, and Dex-BSe at different incubation times. **a, c, d** Data are presented as mean values ± SD (*n* = 3 independent samples). **a, c, d** Statistical significance was calculated by two-tailed Student's *t*-test. Source data are provided as a Source Data file.

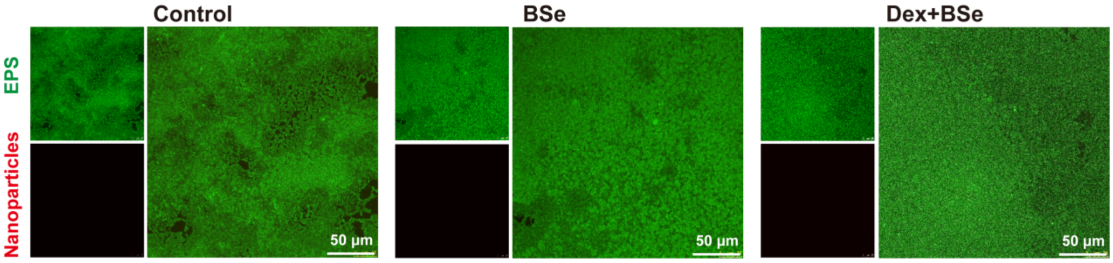

Supplementary Fig. 11 Interactions between nanoparticles with EPS. CLSM images of *S. aureus* biofilms treated with PBS, BSe, and Dex+BSe nanoparticles for 60 min. Green: EPS, Red: Cy5.5-labeled nanoparticles. Experiments were repeated three times independently with similar results.

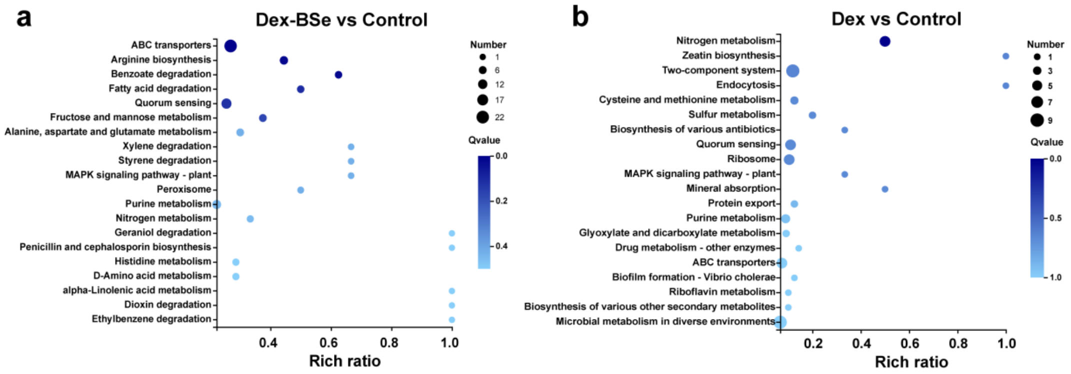

Supplementary Fig. 12 The effect of Dex-BSe and Dex on biofilm-related genes. KEGG enrichment analysis of differentially expressed genes in biofilms after treatment with PBS (Control) and Dex-BSe (**a**), as well as PBS and Dex (**b**), respectively.

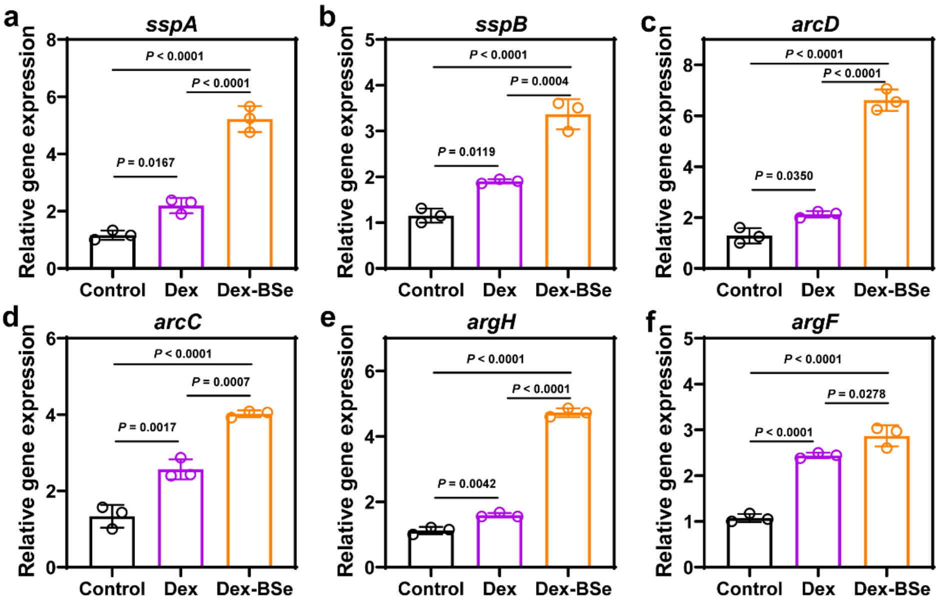

Supplementary Fig. 13 The expression levels of typical genes. RT-PCR results of typical genes encoding extracellular proteases (**a,b**) and involved in arginine biosynthesis and arginine catabolism (**c-f**). Data are presented as mean values ± SD (*n* = 3 independent samples). Statistical significance was calculated by one-way ANOVA using the Tukey post-test. Source data are provided as a Source Data file.

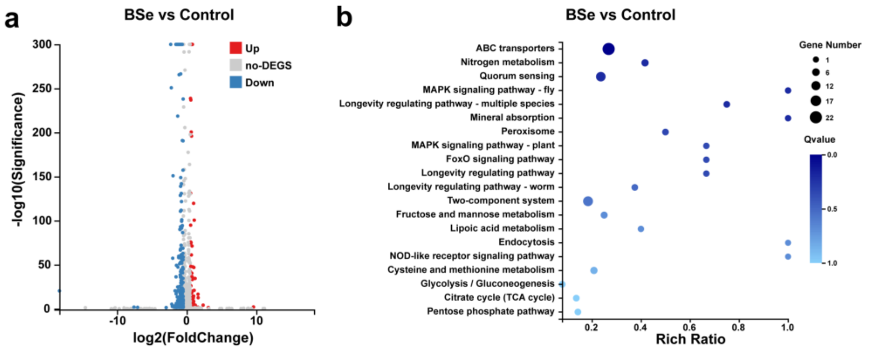

Supplementary Fig. 14 The effect of BSe on biofilm-related genes. a Volcano plot and (**b**) KEGG enrichment analysis of DEGs in biofilms after treatment with PBS (control) and BSe.

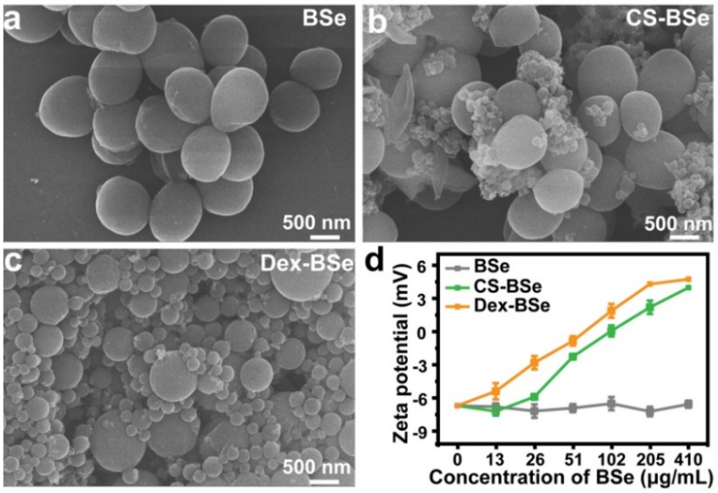

Supplementary Fig. 15 Morphology and zeta potential of *S. aureus* treated with different nanoparticles. SEM images of *S. aureus* treated with (**a**) BSe (205 μg/mL), (**b**) CS-BSe (512 μg/mL), and (**c**) Dex-BSe (512 μg/mL) for 10 min. **d** Zeta potential values recorded for the *S. aureus* dispersions after incubation with different concentrations of BSe, CS-BSe, and Dex-BSe, respectively for 10 min. Data are presented as mean values ± SD (n = 3 independent samples). Source data are provided as a Source Data file.

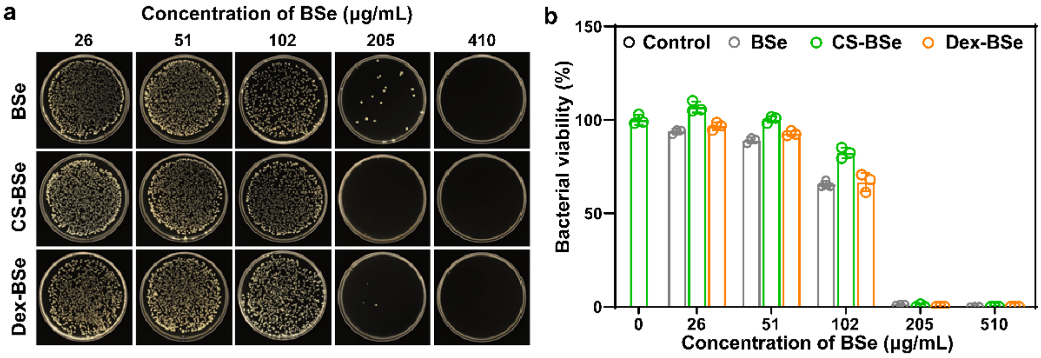

Supplementary Fig. 16 Antibacterial activity of different nanoparticles against *E. coli*. Photographs of *E. coli* colonies (**a**) and the corresponding bacterial viability (**b**) treated with BSe, CS-BSe and Dex-BSe, respectively under 808 nm NIR light irradiaiton (1.0 W/cm^2, 5 min). Data are presented as mean values ± SD (n = 3 independent samples). Source data are provided as a Source Data file.

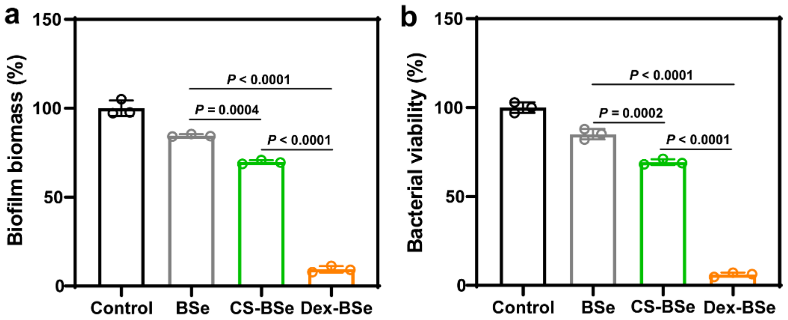

Supplementary Fig. 17 Photothermal killing effect against *S. aureus* biofilms. a Quantitative analysis of the crystal violet-stained *S. aureus* biofilms and (**b**) bacterial viability of *S. aureus* biofilms treated with PBS (control), BSe, CS-BSe and Dex-BSe, respectively under 808 NIR light (1.0 W/cm^2, 5 min) irradiation by standard plate counting method. Data are presented as mean values ± SD (n = 3 independent samples). Statistical significance was calculated by one-way ANOVA using the Tukey post-test. Source data are provided as a Source Data file.

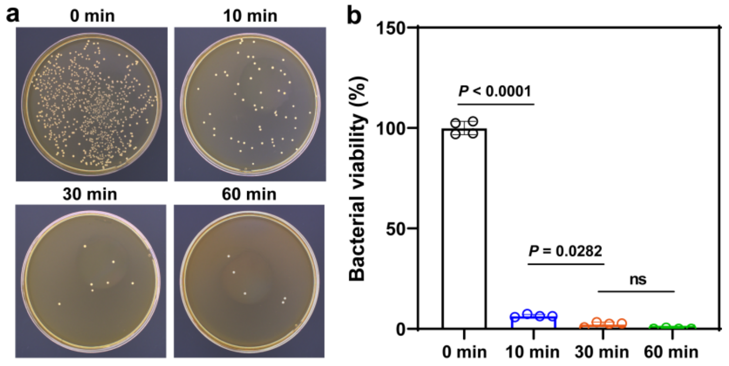

Supplementary Fig. 18 Photothermal antibiofilm effect of Dex-BSe after differet incubation times. a Representative photographs of corresponding *S. aureus* colonies and (**b**) corresponding bacterial viability of *S. aureus* biofilms treated with Dex-BSe (512 μg/mL) under NIR light irradaition (808 nm, 1.0 W/cm^2) for 5 min after different incubation times. Data are presented as mean values ± SD (n = 4 independent samples). Statistical significance was calculated by one-way ANOVA using the Tukey post-test. Source data are provided as a Source Data file.

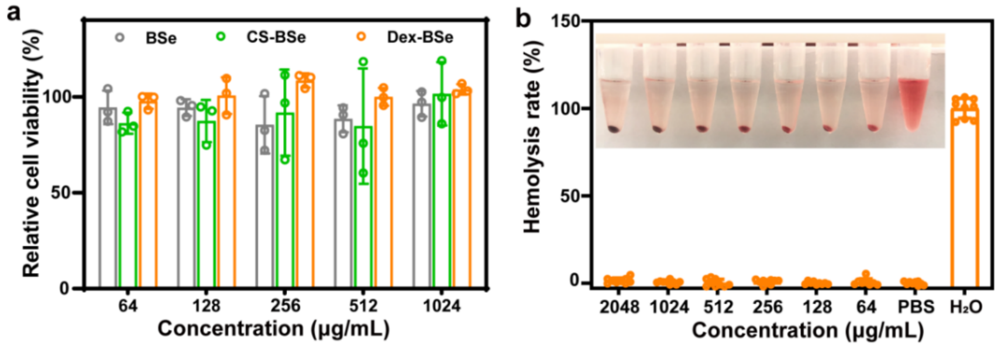

Supplementary Fig. 19 Biocompatibility of Dex-BSe nanoparticles in vitro. a Relative cell viability of L929 cells treated by BSe, CS-BSe and Dex-BSe solutions with different BSe concentrations. Data are presented as mean values ± SD (n = 3 independent samples). **b** Relative hemolysis ratio of Dex-BSe solutions with different concentrations, PBS and H_2O. Insets are the corresponding photographs. Data are presented as mean values ± SD (n = 9 independent samples). Source data are provided as a Source Data file.

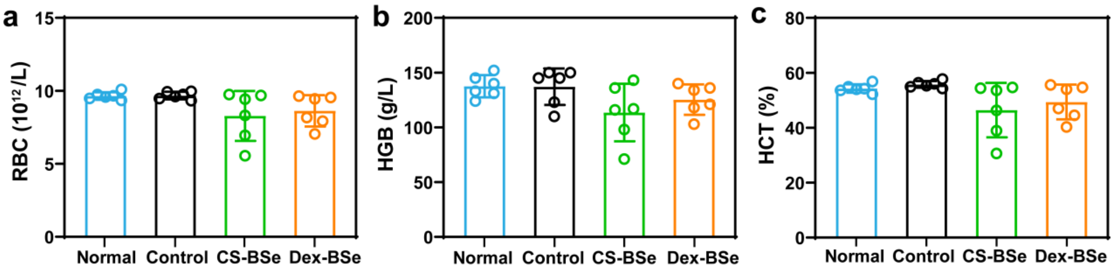

Supplementary Fig. 20 Some blood routine indexes of mice in different groups after 7 days of treatment. The terms are as follows: red blood cells (RBC, **a**), hemoglobin (HGB, **b**), and hematocrit (HCT, **c**). Data are presented as mean values ± SD (n = 6 independent samples). Source data are provided as a Source Data file.

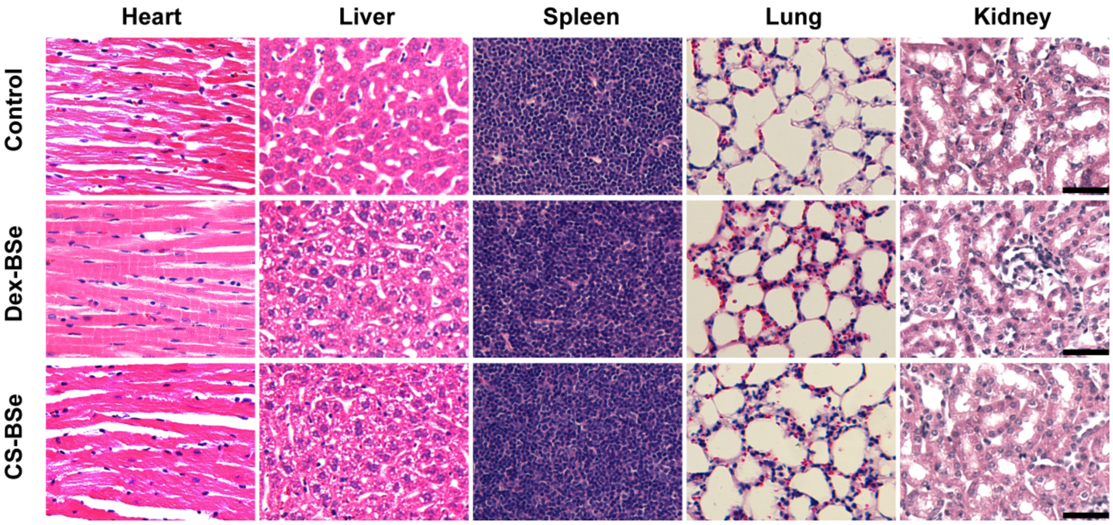

Supplementary Fig. 21 Effect of nanoparticles on major organs. Histology analysis of major organs (heart, liver, spleen, kidney, and lung) of different groups of mice. Scale bar: 50 μm. Experiments were repeated three times independently with similar results.

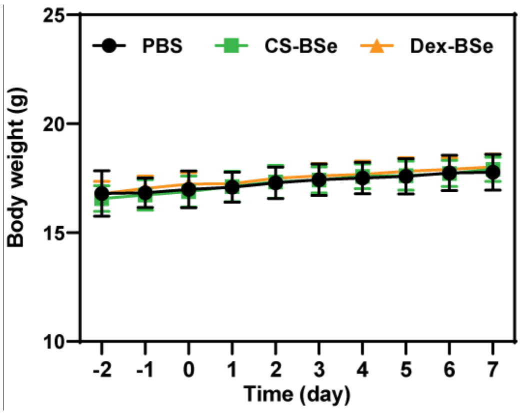

Supplementary Fig. 22 Body weight changes of MRSA-infected mice after different treatments. Data are presented as mean values ± SD (n = 6 independent samples). Source data are provided as a Source Data file.

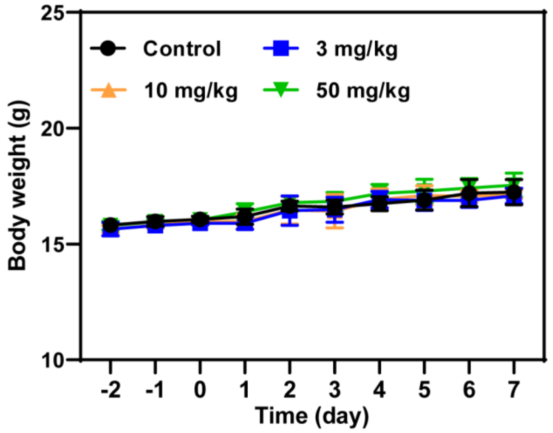

Supplementary Fig. 23. Body weight changes of MRSA-infected mice after treatment with different doses of Dex-BSe nanoparticles. Data are presented as mean values ± SD (n = 5 independent samples). Source data are provided as a Source Data file.

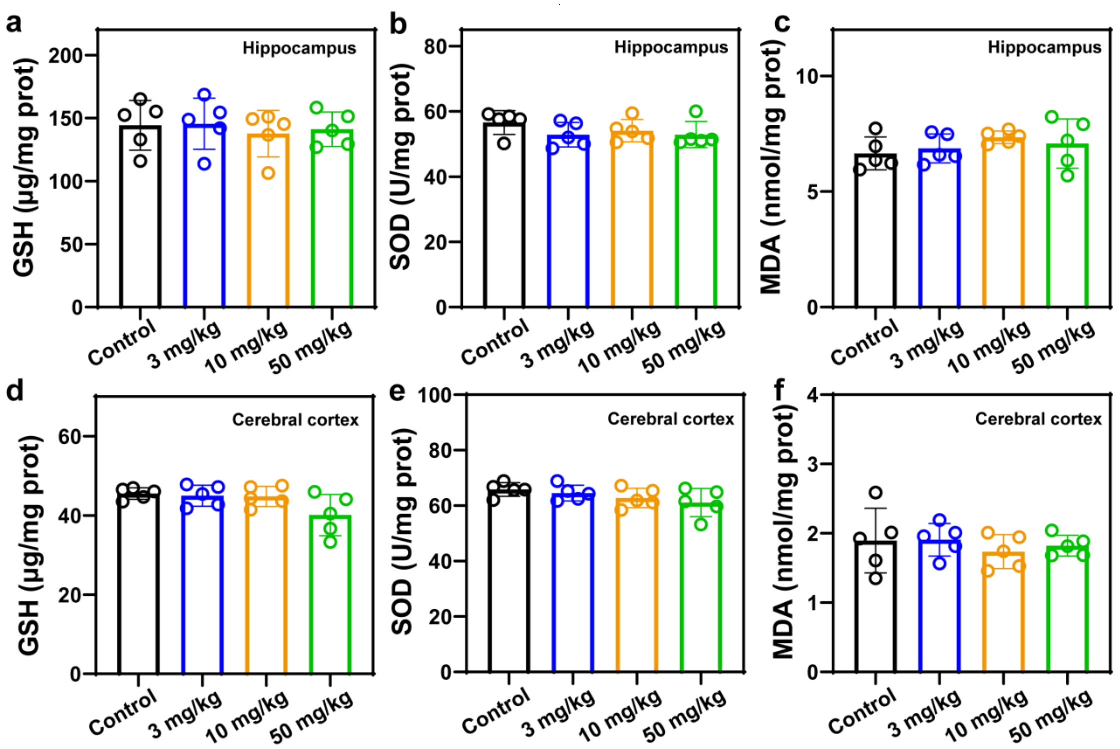

Supplementary Fig. 24 Effects of different doses of Dex-BSe on the oxidative stress in the central nervous system. The activity of (**a**) glutathione (GSH) and (**b**) superoxide dismutase (SOD), and (**c**) the malondialdehyde (MDA) level in hippocampus treated with different doses of Dex-BSe after 7 days. The activity of (**d**) GSH and (**e**) SOD, and (**f**) the MDA level in cerebral cortex after treatment with different doses of Dex-BSe after 7 days. Data are presented as mean values ± SD (n = 5 independent samples). Source data are provided as a Source Data file.

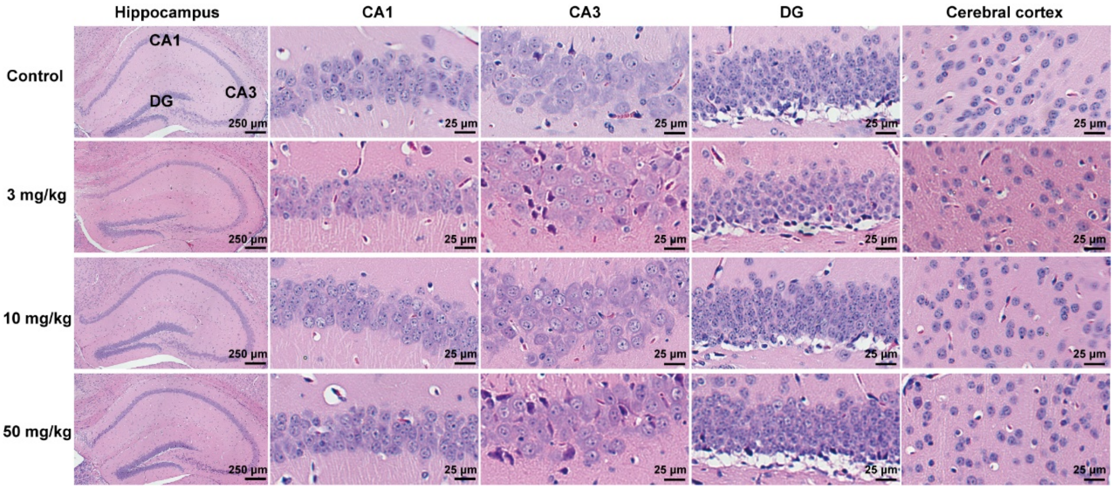

Supplementary Fig. 25 Effects of different doses of Dex-BSe on neurons. H&E staining images of hippocampus and cerebral cortex of mice treated with different doses of Dex-BSe after 7 days. Experiments were repeated three times independently with similar results.

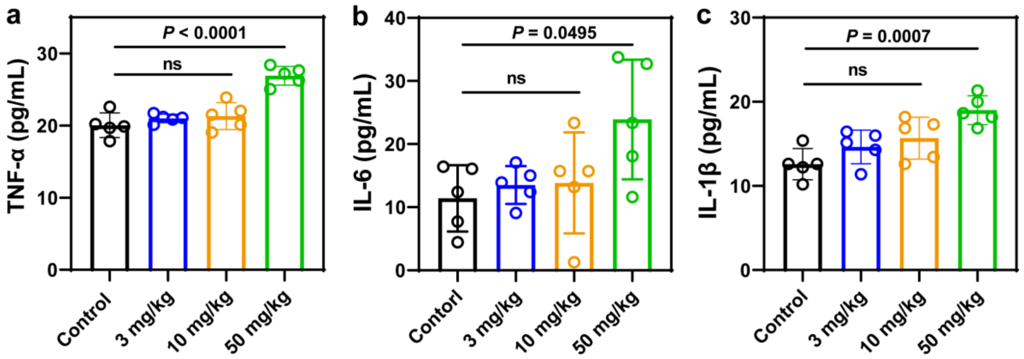

Supplementary Fig. 26 Effects of different doses of Dex-BSe on pro-inflammatory cytokines in bronchoalveolar lavage fluid. The levels of TNF-α (**a**), IL-6 (**b**) and IL-1β (**c**) in bronchoalveolar lavage fluid after different treatments. Data are presented as mean values ± SD (n = 5 independent samples). Statistical significance was calculated by one-way ANOVA using the Tukey post-test. Source data are provided as a Source Data file.

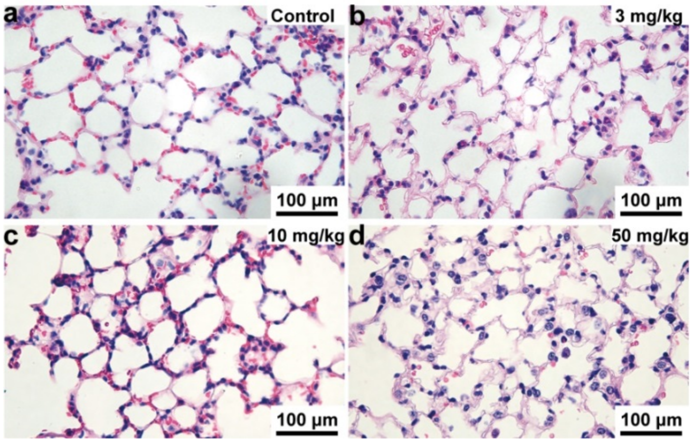

Supplementary Fig. 27 Effects of different doses of Dex-BSe on lung tissues. H&E staining images of lung tissues of mice in the control group (**a**) and treated with different doses of Dex-BSe (**b**) 3 mg/kg, (**c**) 10 mg/kg, and (**d**) 50 mg/kg. **a-d** Experiments were repeated three times independently with similar results.

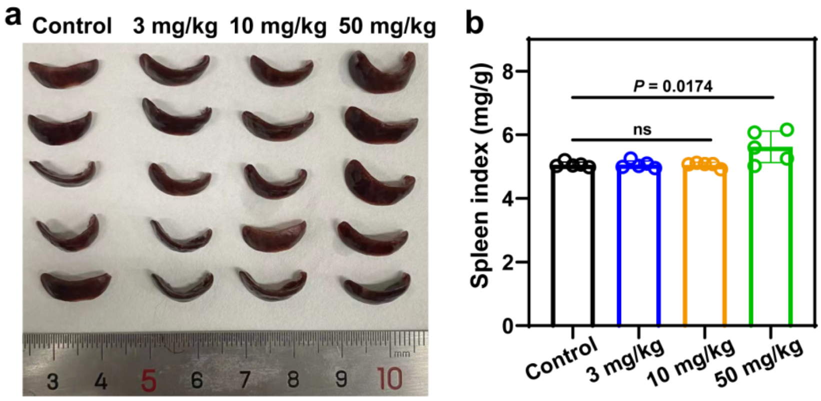

Supplementary Fig. 28 Effects of different doses of Dex-BSe on spleen. a Photograph of spleens collected from mice after 7 days of treatment with different doses of Dex-BSe. **b** Quantification of the corresponding spleen/body weight. Data are presented as mean values ± SD (*n* = 5 independent samples). Statistical significance was calculated by one-way ANOVA using the Tukey post-test. Source data are provided as a Source Data file.

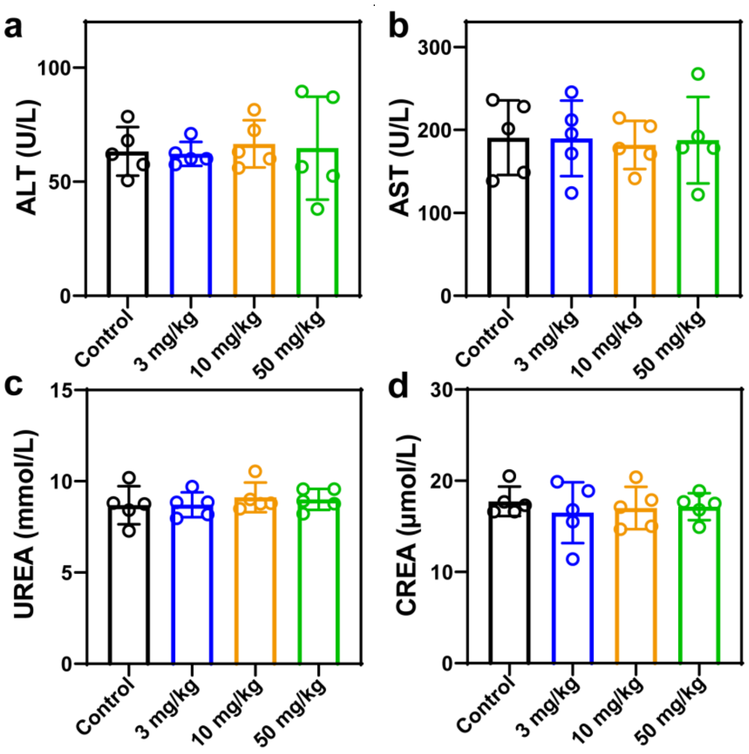

Supplementary Fig. 29 Effects of different doses of Dex-BSe on liver and kidney function. Kidney and liver function markers of mice after 7 days of treatment with different doses of Dex-BSe. **a** ALT: alanine aminotransferase; **b** AST: aspartate aminotransferase; **c** UREA: urea nitrogen; and **d** CREA: creatinine. Data are presented as mean values ± SD (*n* = 5 independent samples). Source data are provided as a Source Data file.

Supplementary Table 1. Primer sequences

Primer name	Sequence (5' to3')
sspA forward	TGCCAACGATGACCAACCTA
sspA reverse	CATTGTCTGGATTGTCTGGATTATC
sspB forward	GAAGCGATACAAGAAGATCAAGTTC
sspB reverse	TAATGCTGCCATACTGAATCCTG
arcC forward	GGTATGATAGGCTATTGGTTGGAA
arcC reverse	GGTTTGGTTGGGTTATTGAATCG
arcD forward	TTCTGCTGAATGACACCTTGG
arcD reverse	TAGGAACTGCTACCGTTATTGG
argH forward	ACTTACCACCAGCATCACCAA
argH reverse	GCGAATCAAGGCATTATTAGTCAAC
argF forward	GATGGCATTGAATACCGTGGTT
argF reverse	CAGCAAGAACTTGAGTAGGATGAT
16S forward	GTCTTGCTGTCACTTATAGATGGA
16S reverse	GTTGCCTTGGTAAGCCGTTA

Supplementary References

1. Li, L., Cao, R., Wang, Z., Li, J. & Qi, L. Template synthesis of hierarchical Bi_2E_3 (E = S, Se, Te) core−shell microspheres and their electrochemical and photoresponsive properties. *J. Phys. Chem. C* **113**, 18075−18081 (2009).
2. Xie, H. et al. Metabolizable ultrathin Bi_2S_3 nanosheets in imaging-guided photothermal therapy. *Small* **12**, 4136−4145 (2016).
3. Liu, Y., Zhao, N. & Xu, F. J. pH-responsive degradable dextran-quantum dot nanohybrids for enhanced gene delivery. *ACS Appl. Mater. Interfaces* **11**, 34707−34716 (2019).
4. Roper, D. K., Ahn, W. & Hoepfner, M. Microscale heat transfer transduced by surface plasmon resonant gold nanoparticles. *J. Phys. Chem. C* **111**, 3636−3641 (2007).
