# Supplementary material for: Janus nanoparticles targeting extracellular polymeric substance achieve flexible elimination of drug-resistant biofilms
Source: Nat Commun. 2023 Aug 23;14:5132. doi: 10.1038/s41467-023-40830-9 (PMC10447547; doi:10.1038/s41467-023-40830-9)
Supplement: Supplementary file 4 — Description of Additional Supplementary Files [file 41467_2023_40830_MOESM4_ESM.pdf]

Title: Supplementary Movie 1

Description: The trajectory tracking of Dex-BSe nanoparticles in the absence and presence of NIR irradiation.
